# The Changing Face of *in vitro* Culture Models for Thyroid Cancer Research: A Systematic Literature Review

**DOI:** 10.3389/fsurg.2020.00043

**Published:** 2020-07-16

**Authors:** Dylan Chew, Victoria Green, Andrew Riley, Richard James England, John Greenman

**Affiliations:** ^1^Department of ENT, Hull University Teaching Hospitals NHS Trust, Castle Hill Hospital, London, United Kingdom; ^2^Department of Biomedical Sciences, University of Hull, Hull, United Kingdom

**Keywords:** thyroid cancer, *in vitro*, thyrocyte, organoids, epigenetics, drugs, cancer stem cells

## Abstract

**Background:** Thyroid cancer is the most common endocrine malignancy worldwide. Primary treatment with surgery and radioactive iodine is usually successful, however, there remains a small proportion of thyroid cancers that are resistant to these treatments, and often represent aggressive forms of the disease. Since the 1950s, *in vitro* thyroid culture systems have been used in thyroid cancer research. *In vitro* culture models have evolved from 2-dimensional thyrocyte monolayers into physiologically functional 3-dimensional organoids. Recently, research groups have utilized *in vitro* thyroid cancer models to identify numerous genetic and epigenetic factors that are involved with tumorigenesis as well as test the efficacy of cytotoxic drugs on thyroid cancer cells and identify cancer stem cells within thyroid tumors.

**Objective of Review:** The objective of this literature review is to summarize how thyroid *in vitro* culture models have evolved and highlight how *in vitro* models have been fundamental to thyroid cancer research.

**Type of Review:** Systematic literature review.

**Search Strategy:** The National Institute for Health and Care Excellence (NICE) Healthcare and Databases Advanced Search (HDAS) tool was used to search EMBASE, Medline and PubMed databases. The following terms were included in the search: “*in vitro*” AND “thyroid cancer”. The search period was confined from January 2008 until June 2019. A manual search of the references of review articles and other key articles was also performed using Google Scholar.

**Evaluation Method:** All experimental studies and review articles that explicitly mentioned the use of *in vitro* models for thyroid cancer research in the title and/or abstract were considered. Full-text versions of all selected articles were evaluated. Experimental studies were reviewed and grouped according to topic: genetics/epigenetics, drug testing/cancer treatment, and side populations (SP)/tumor microenvironment (TME).

**Results:** Three thousand three hundred and seventy three articles were identified through database and manual searches. One thousand two hundred and sixteen articles remained after duplicates were removed. Five hundred and eighty nine articles were excluded based on title and/or abstract. Of the remaining 627 full-text articles: 24 were review articles, 332 related to genetic/epigenetics, 240 related to drug testing/treatments, and 31 related to SP/TME.

**Conclusion:** *In vitro* cell culture models have been fundamental in thyroid cancer research. There have been many advances in culture techniques- developing complex cellular architecture that more closely resemble tumors *in vivo*. Genetic and epigenetic factors that have been identified using *in vitro* culture models can be used as targets for novel drug therapies. In the future, *in vitro* systems will facilitate personalized medicine, offering bespoke treatments to patients.

## Introduction

Thyroid cancers are the most common endocrine malignancies worldwide (1). In most developed countries the incidence of thyroid cancer has been steadily rising, partially attributed to an increased diagnosis of subclinical papillary micro-carcinomas (2). Despite its prevalence, the overall mortality rate of thyroid cancer has remained low (0.5 per 100,000 patients) (3). Surgery followed by radioactive-iodine (RAI) therapy continue to be the first line treatment modalities for thyroid cancer. Overall survival rates following primary treatment are high (>98% 5-year survival), however, for the 1–2% of patients with aggressive forms of the disease or the 5–10% of patients with distant metastases, the prognosis is far worse (4).

Thyroid cancer research is focused on improving our understanding of the biological mechanisms that initiate and propagate the disease in the hope of refining diagnoses and formulating bespoke treatments to improve patient outcomes. An essential foundation of this research is the use of *in vitro* experimental models. In the simplest terms, an *in vitro* culture model is comprised of a vessel (e.g., dish, plate, or well) containing a culture medium to support and maintain cells outside of the body for experimental purposes. Culture models have evolved from growing homogenous cell populations in a 2-dimensional (2D) monolayer into complex 3-dimensional (3D) heterogeneous multicellular structures that resemble tissues *in vivo*. Cells used in these models can be derived from immortalized cell lines, pluripotent stem cells or *ex-vivo* human tissue. Individual patients' explanted thyroid tissue can be maintained *in vitro* for several days using microfluidic technology, an advancement which will open the gateway for personalized cancer medicine (5). This review summarizes how *in vitro* culture models have evolved and how they have been applied to thyroid cancer research.

## Methods

### Search Strategy

The National Institute for Health and Care Excellence (NICE) Healthcare and Databases Advanced Search (HDAS) tool was used to search EMBASE, Medline and PubMed databases. The following terms were included in the search: “*in vitro*” and “thyroid cancer”. The search period was confined from January 2008 until June 2019. A manual search of the references of review articles and other key articles was also performed using Google Scholar.

### Article Selection

**Figure 1:**
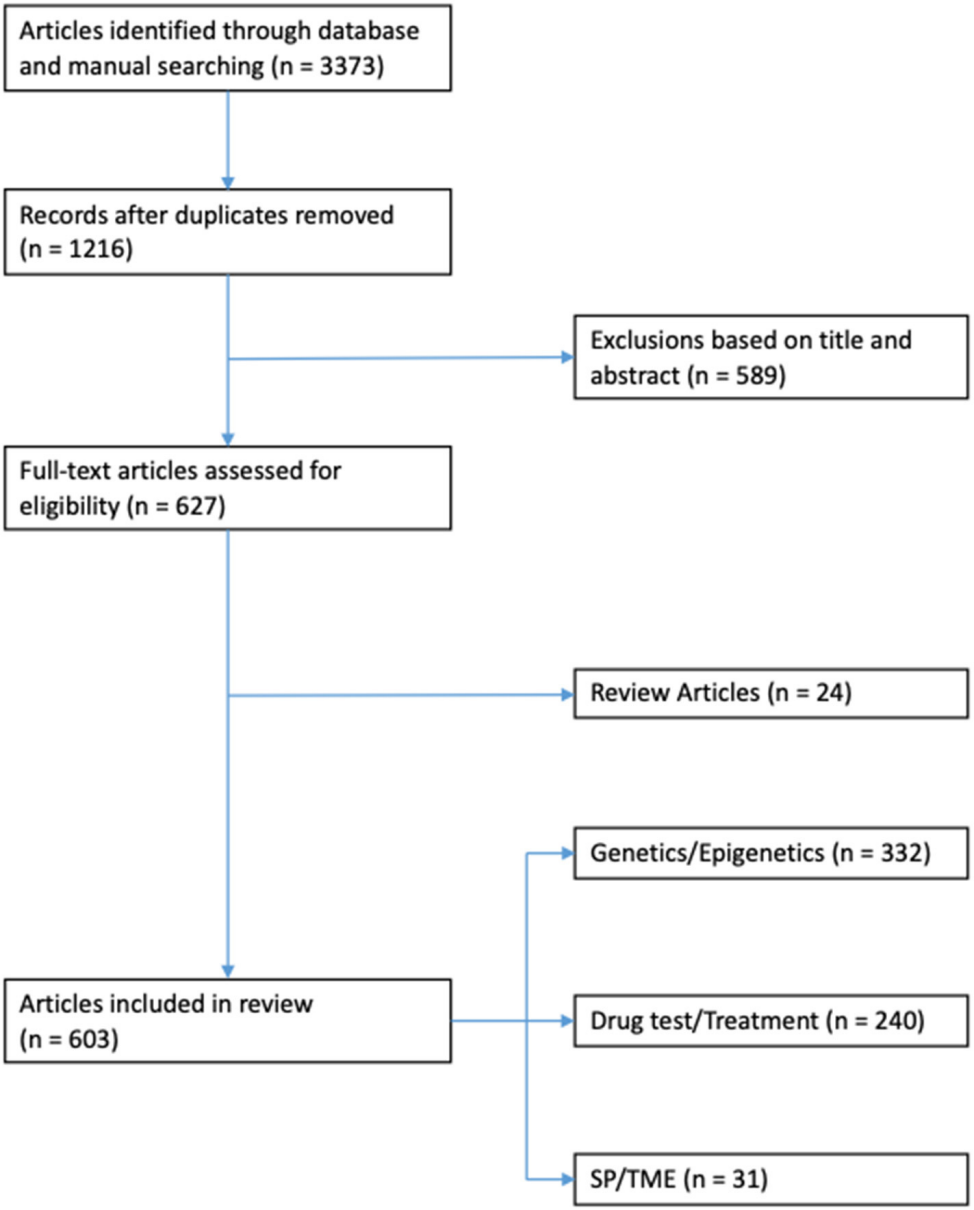
Flowchart of article selection based on PRISMA guidelines.

**Figure 2:**
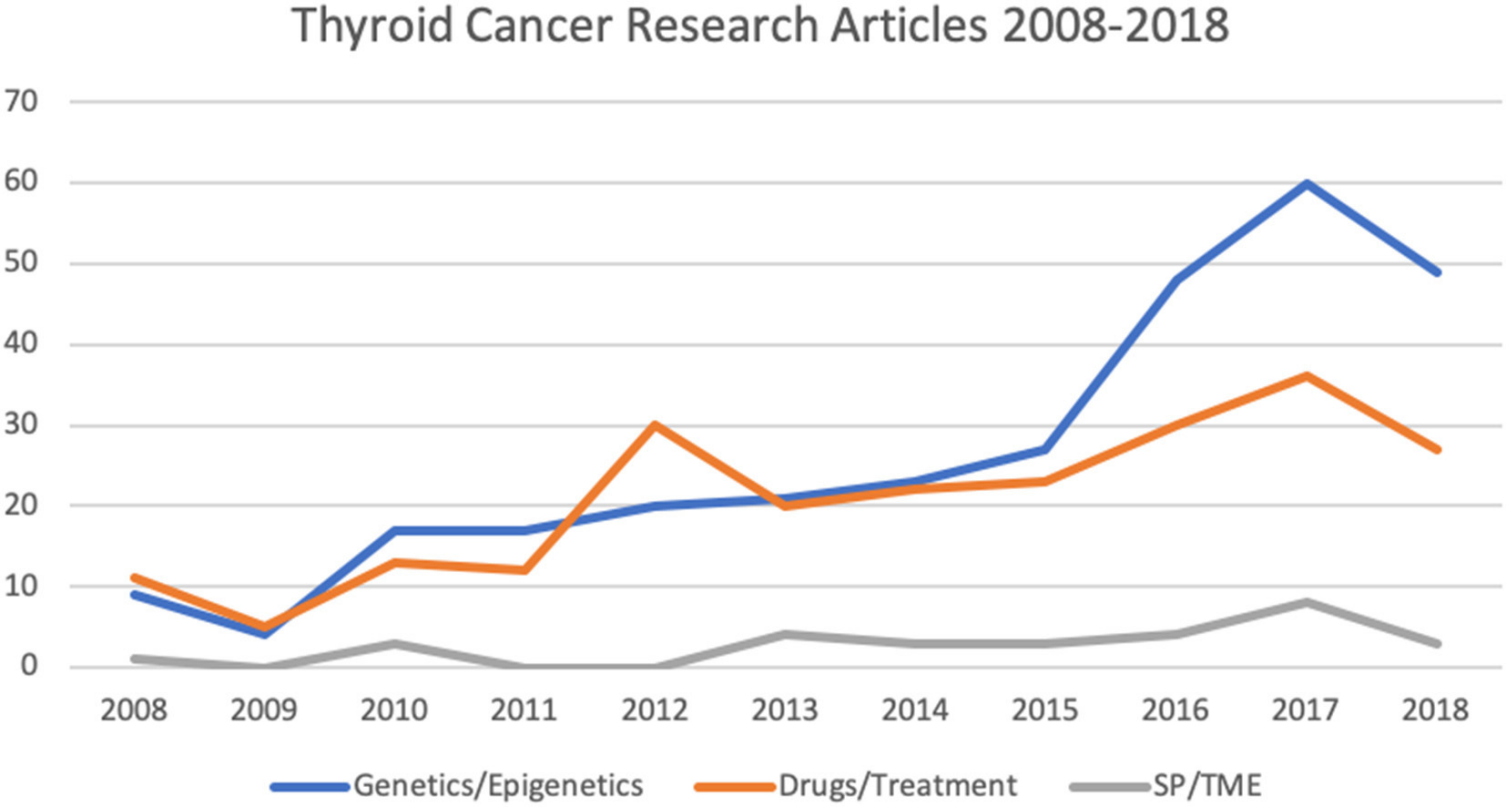
Number of articles relating to thyroid cancer research involving *in vitro* culture systems published from 2008 to 2018 (as per HDAS search on 19 January 2019). SP, side populations; TME, tumor microenvironment.

All experimental studies and review articles that explicitly mentioned the use of *in vitro* models for thyroid cancer research in the title and/or abstract were considered (Figure 1). Full-text versions of all selected articles were evaluated. Experimental studies were reviewed and grouped according to topic: genetics/epigenetics, drug testing/cancer treatment and side populations (SP)/tumor microenvironment (TME; Figure 2).

## The Evolution of Cell Culture Models

### 2D vs. 3D

**Figure 3:**
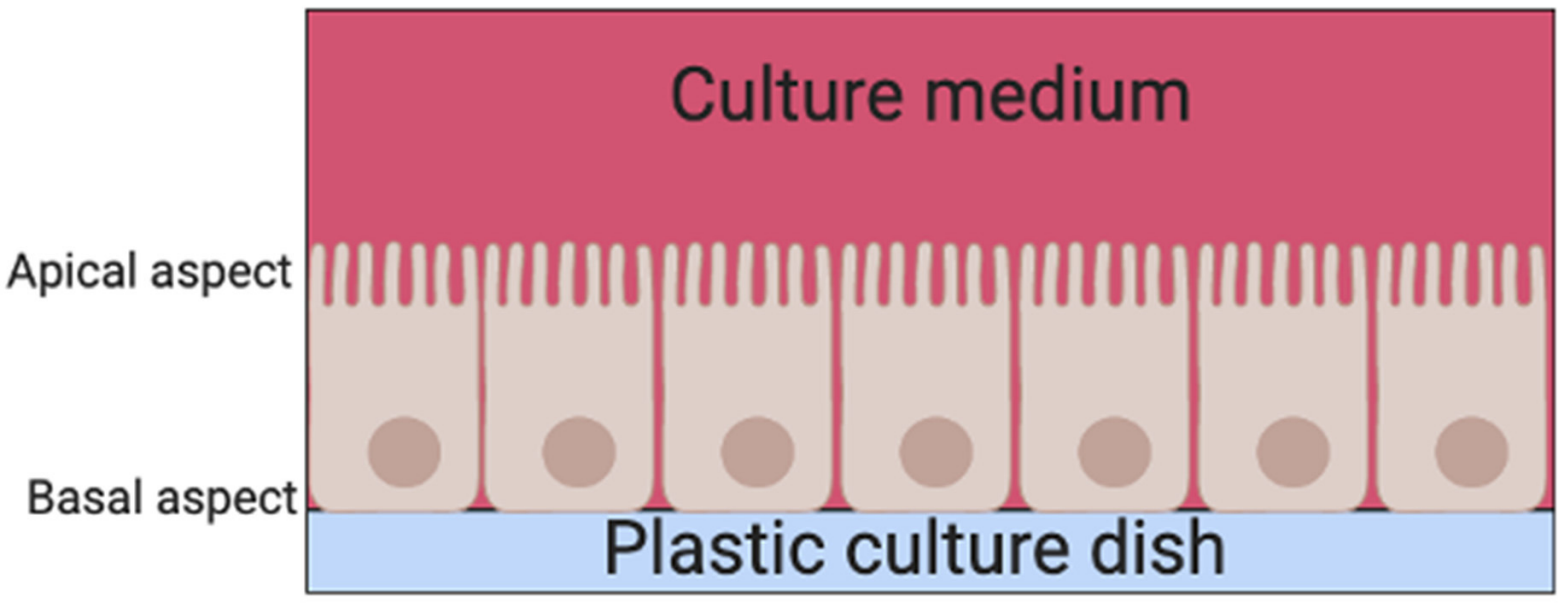
A schema of thyrocytes in a 2D monolayer culture system.

There are two basic systems for growing cells in culture, as a single layer of cells on an artificial substrate (adherent culture) or free-floating in the culture medium (suspension culture). Thyrocyte 2D monolayer culture systems have been used since the late 1950s (6). Their main limitation is that thyrocytes are unable to arrange themselves into their normal physiological follicular structures when cultured on adherent plates in standard culture medium (7). Instead, thyrocytes are arranged into a continuous epithelial sheet, with the apical aspect of the cell facing the culture medium above and the basal aspect facing the surface of the dish (Figure 3).

**Figure 4:**
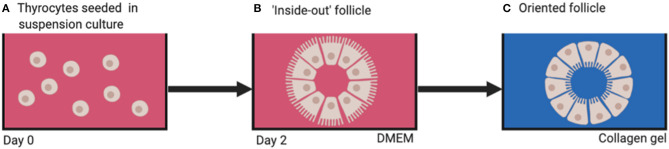
**(A)** Dissociated thyrocytes placed in suspension culture, **(B)** “inside-out” follicles form after 2 days, **(C)** physiologically oriented follicles form when embedded in ECM substrate (e.g., type 1 collagen). DMEM, Dulbecco's modified eagle's medium.

When thyrocytes are suspended in non-adherent vessels containing culture medium, they arrange themselves into a follicular structure (Figure 4A). However, the orientation of the cells is such that the apical aspect with microvilli are facing outwards in contact with the culture medium (7) (Figure 4B), thus creating an “inside-out” follicle. If these inside-out follicles are then embedded into a 3D substrate emulating thyroid extracellular matrix (ECM; e.g., type 1 collagen gel) the cellular polarity inverts with the microvilli facing inwards toward the follicular lumen, creating a true physiological thyroid follicle (Figure 4C). This leads to the conclusion that thyroid folliculogenesis is dependent on the presence of ECM.

Such collagen gel cultures were developed in the 1970s to enable reconstruction of thyroid follicles *in vitro* (8). These 3D culture systems have not only allowed study of thyroid folliculogenesis but also thyroid function under stimulation by factors such as thyroid stimulating hormone (TSH) and iodine, as well as interactions between thyrocytes and the ECM (9).

By replicating the *in vivo* thyroid cellular structure, 3D cell culture systems allow researchers to study the complex spatial morphology that facilitates cell-cell and cell-matrix interactions and signaling—a huge advantage over 2D monolayer culture systems (10). Advances in cell biology, microfabrication and tissue engineering have facilitated development of a wide range of 3D cell culture techniques including spheroids, organoids and microfluidic systems (11).

### Spheroids

**Figure 5:**
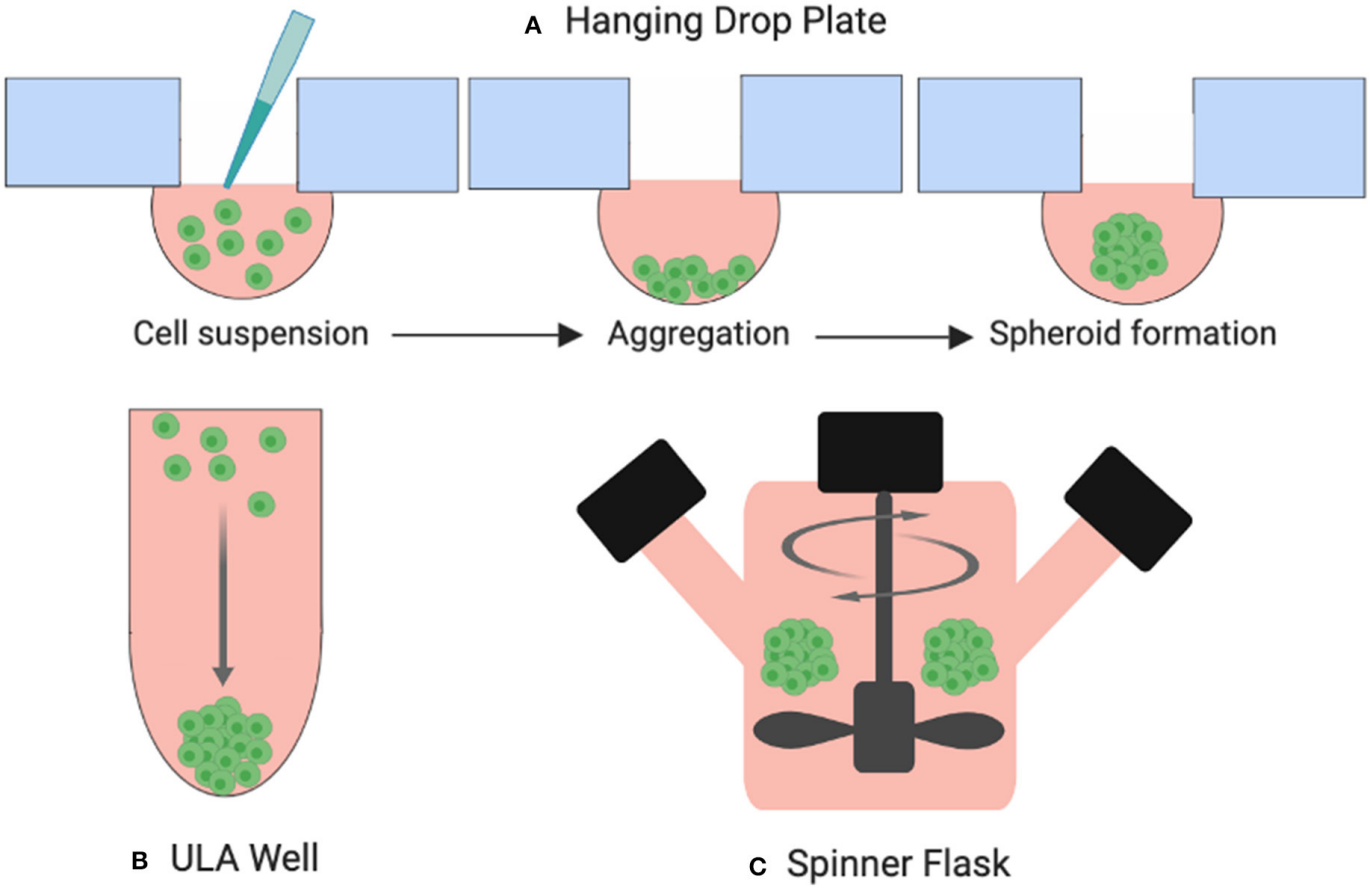
Three examples of spheroid formation techniques. **(A)** Cells suspended in droplets attached to hanging drop plates **(B)** Ultra-low attachment (ULA) plates prevent cells from adhering to the surface of the wells forcing them to aggregate and form spheroids, **(C)** cells suspended in a spinner flask are stirred by an element producing large yields of spheroids.

One of the most common 3D culture models used in thyroid cancer research today is the multicellular spheroid. Spheroids are cellular aggregates consisting of several thousand phenotypically distinct cells. A technique for developing spheroids was pioneered by Sutherland et al. in the early 1970s for testing the response of radiation exposure on tumor cell lines (12). The resulting dose response curves were similar to those produced when irradiating *ex-vivo* solid animal tumors. Since then, several techniques to create spheroids have been established including hanging drop plates, low-adhesion surface methods, and suspension culture in bioreactors which drive cells to self-aggregate under dynamic conditions (Figure 5). Spheroids can be grown to a variety of sizes, depending on the needs of the study (13). As well as thyrocytes, these models have been developed to include co-culture with immune cells such as macrophages (14) and neutrophils (15).

**Figure 6:**
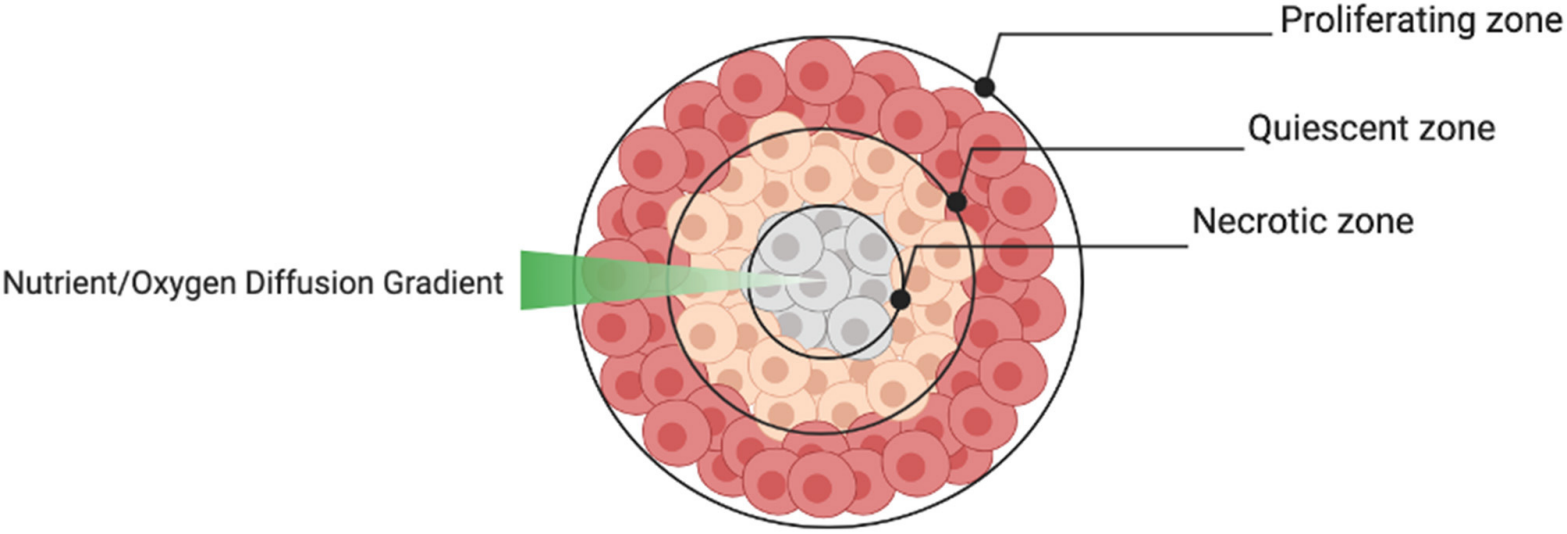
Schematic of cellular strata within a spheroid.

The aggregate structure of 3D spheroids supersedes 2D monolayers in terms of their ability to reproduce the cellular heterogeneity of tumors *in vivo*. Depending on the size of the spheroid, the structure usually consists of an outer layer of proliferating cells, a middle layer of quiescent cells and a central core of necrotic cells caused by a nutrient and oxygen diffusion gradient (Figure 6). This structural heterogeneity is important to consider when spheroids are used to test drug sensitivity.

Despite their many advantages, spheroids do present some practical challenges. Firstly, it can be difficult establishing spheroids from a small seed number of cells. Also, controlling proliferation, specific ratios of various co-cultured cell types and maintaining spheroids of a uniform size is not always achievable (16). This leads to issues with standardizing culture and assay protocols as well as evaluating output data (17). Currently there is no reliable, standardized high-throughput assay that allows spheroid use for drug screening. Furthermore, despite having the ability to co-culture thyroid cancer cells with select immune cells, spheroids do not entirely mimic the TME in terms of representing all the cell types present *in vivo*.

### Organoids

**Figure 7:**
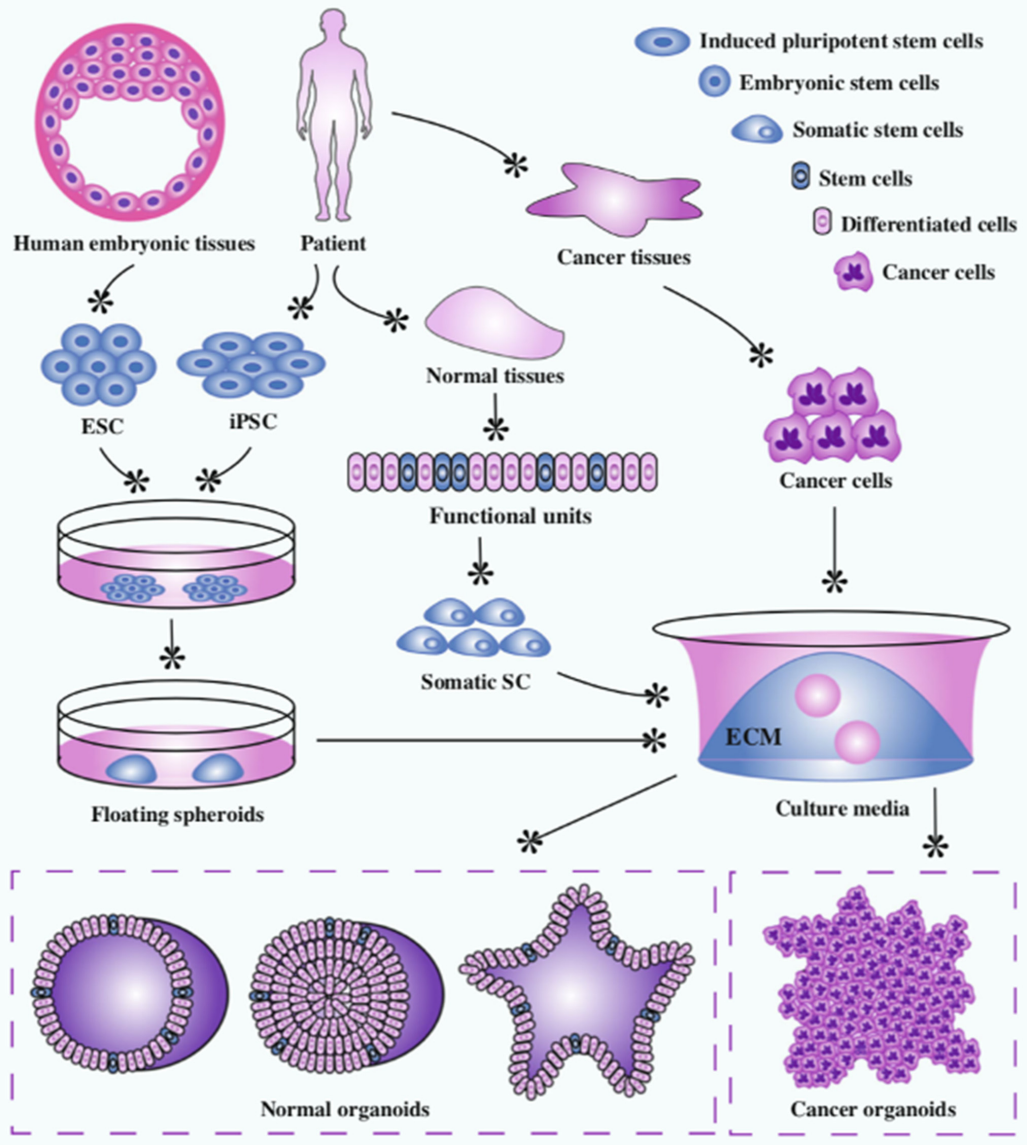
Organoid development from stem cells, primary tissue, and cancer cell.

Organoids are 3D *in vitro* cellular structures derived from either embryonic stem cells (ESC), induced pluripotent stem cells (iPSC), organ-specific adult stem cells (ACS), or primary cancer cells (18) (Figure 7). Organoids are defined by three characteristics: self-organization, multicellularity, and functionality (19). The constituent cells of an organoid are arranged into a 3D structure characteristic of the organ *in vivo*. They generally contain all the cell types found within that organ and execute the same functions they would normally carry out.

Novel culture systems containing laminin-rich Matrigel as a substitute for ECM and growth factors including EGF, Noggin, Wnt, and R-spondin allow the development of organoids from stem cells (20). The *in vitro* process utilizes the defining characteristics of stem cells: namely, the clonal expansion capacity and production of daughter cells that can differentiate into multiple cell types (21). It then relies on cell-cell and cell-matrix interaction and signaling to form the organoid structure.

Organoids have been applied to understand stem cell biology, organogenesis and pathogenesis of various diseases (10, 18, 20–26). Organoids have huge potential for modeling cancer and many organoid systems such as breast (27), colorectal (28), and prostate (29) have already been established in experimental studies. In 2018, Saito et al., established a thyroid organoid culture system from murine stem cells (25). These organoids successfully functioned as thyroid tissue, producing thyroglobulin and thyroid hormone (T3) when exposed to thyroid stimulating hormone (TSH). After p53 knockout, these organoids were xenografted into recipient mice which subsequently developed poorly differentiated thyroid cancer. Presently this is the only published study that has established a thyroid organoid as a novel experimental model.

Although organoids closely represent the cellular structure and function of *in vivo* tissues, there are still limitations—they often only demonstrate the initial stages of organogenesis/tumorigenesis, they lack the full range of cells that exist in the TME, and they do not develop tissue support structures such as a vascular or neuronal network (11).

### Microfluidic Systems

Microfluidic systems (MFS) are devices that maintain and analyze small *ex-vivo* tissue samples or 3D cultured cells in a pseudo-*in vivo* state (30). The basic design is made up of a “chip” which houses the tissue sample connected to inlet and outlet tubing for circulating fluids. MFS mimic the human body's vasculature and lymphatics through continuous perfusion of nutrients via micro-volumes of fluid while simultaneously removing waste products. A significant advantage of maintaining tissue in these devices is that the cells remain viable and maintain tissue architecture for longer periods (3-7 days) than conventional *in vitro* culture systems (31).

MFS have been used to interrogate numerous types of cancer such as breast (32), lung (33), head, and neck squamous cell carcinoma (31), and only very recently thyroid cancer (5). These devices offer the ability to test drugs and RAI sensitivity of individual thyroid tumors to potentially customize/personalize therapeutic regimes.

## Recent Application of *In Vitro* Culture Systems in Thyroid Cancer Research

### Genetics and Epigenetics

In the past ten years there have been over 300 published papers utilizing *in vitro* models to study the molecular biology of thyroid cancer. Mutations of genes such as *RET, BRAF* and *RAS* are widely recognized as contributing to thyroid carcinogenesis (34). These mutations lead to uncontrolled cellular proliferation, de-differentiation and metastasis through signaling pathways such as mitogen-activated protein kinase (MAPK), phosphoinositide 3-kinase (PI3K)/Akt, and Wnt/β-catenin (35). Epigenetic factors, including messenger RNA (mRNA), micro RNA (miRNA) and long non-coding RNA (lncRNA), control gene expression through mechanisms such as DNA methylation and histone modification. Epigenetic factors can be over-expressed or under-expressed in thyroid cancers and activate the same signaling pathways mentioned above (Table 1).

**Table 1:** Studies related to genetics and epigenetics in thyroid cancer research published from Jan 2018—Jun 2019 (as per HDAS search on 19 July 2019).

**Publication title**	**References**	**Models used**
Estrogen receptor B upregulated by IncRNA-H19 to promote cancer stem-like properties in papillary thyroid carcinoma	(36)	2D, spheroids and PDX^*^
MicroRNA-125b Interacts with Foxp3 to Induce Autophagy in Thyroid Cancer	(37)	2D
Long non-coding RNA UCA1 promotes papillary thyroid cancer cell proliferation via miR-204-mediated BRD4 activation	(38)	2D and PDX
MiR-26a inhibits thyroid cancer cell proliferation by targeting ARPP19	(39)	2D and PDX
Long Noncoding RNA LINC003121 Inhibits Proliferation and Invasion of Thyroid Cancer Cells by Suppression of the Phosphatidylinositol-3-Kinase (PI3K)/Akt Signaling Pathway	(40)	2D
Long Non-coding Antisense RNA TNRC6C-AS1 Is Activated in Papillary Thyroid Cancer and Promotes Cancer Progression by Suppressing TNRC6C Expression	(41)	2D
miR-214 regulates papillary thyroid carcinoma cell proliferation and metastasis by targeting PSMD10	(42)	2D
DNA copy number gain-mediated lncRNA LINC01061 upregulation predicts poor prognosis and promotes papillary thyroid cancer progression	(43)	2D and PDX
Upregulated hsa_circ_0004458 Contributes to Progression of Papillary Thyroid Carcinoma by Inhibition of miR-885-5p and Activation of RAC1	(44)	2D and PDX
UHRF1 suppression promotes cell differentiation and reduces inflammatory reaction in anaplastic thyroid cancer	(45)	2D, spheroids and PDX
Long noncoding RNA UCA1 promotes anaplastic thyroid cancer cell proliferation via miR-135a-mediated c-myc activation	(46)	2D and PDX
miR-329 inhibits papillary thyroid cancer progression via direct targeting WNT1	(47)	2D and PDX
miR-129 regulates growth and invasion by targeting MAL2 in papillary thyroid carcinoma	(48)	2D and PDX
miR-218 overexpression suppresses tumorigenesis of papillary thyroid cancer via inactivation of PTEN/PI3K/AKT pathway by targeting Runx2	(49)	2D and PDX
UCA1 promotes papillary thyroid carcinoma development by stimulating cell proliferation via Wnt pathway	(50)	2D
SDC4 Gene Silencing Favors Human Papillary Thyroid Carcinoma Cell Apoptosis and Inhibits Epithelial Mesenchymal Transition via Wnt/β-Catenin Pathway	(51)	2D
c-Myc Is a Major Determinant for Antitumor Activity of Aurora A Kinase Inhibitor MLN8237 in Thyroid Cancer	(52)	2D
CircNUP214 sponges miR-145 to promote the expression of ZEB2 in thyroid cancer cells	(53)	2D and PDX
CLDN10 is Associated with Papillary Thyroid Cancer Progression	(54)	2D
LncRNA XIST/miR-34a axis modulates the cell proliferation and tumor growth of thyroid cancer through MET-PI3K-AKT signaling	(55)	2D and PDX
CITED1 promotes proliferation of papillary thyroid cancer cells via the regulation of p21 and p27	(56)	2D and PDX
Long Noncoding RNA HOXA-AS2 Promotes Papillary Thyroid Cancer Progression by Regulating miR-520c-3p/S100A4 Pathway	(57)	2D and PDX
KLF5 promotes the tumorigenesis and metastatic potential of thyroid cancer cells through the NF-κB signaling pathway	(58)	2D and PDX
TEKT4 Promotes Papillary Thyroid Cancer Cell Proliferation, Colony Formation, and Metastasis through Activating PI3K/Akt Pathway	(59)	2D
Downregulation of MiR-431 expression associated with lymph node metastasis and promotes cell invasion in papillary thyroid carcinoma	(60)	2D
Long noncoding RNA LINC00313 modulates papillary thyroid cancer tumorigenesis via sponging miR-4429	(61)	2D
Long non-coding RNA BANCR regulates cancer stem cell markers in papillary thyroid cancer via the RAF/MEK/ERK signaling pathway	(62)	2D, spheroids and PDX
LRP4 promotes proliferation, migration, and invasion in papillary thyroid cancer	(63)	2D
MicroRNA-222 Promotes Invasion and Metastasis of Papillary Thyroid Cancer Through Targeting Protein Phosphatase 2 Regulatory Subunit B Alpha Expression	(64)	2D and PDX
Circular RNA circZFR contributes to papillary thyroid cancer cell proliferation and invasion by sponging miR-1261 and facilitating C8orf4 expression	(65)	2D
Long noncoding RNA PVT1 enhances the viability and invasion of papillary thyroid carcinoma cells by functioning as ceRNA of microRNA-30a through mediating expression of insulin like growth factor 1 receptor	(66)	2D
Inhibitory roles of miR-9 on papillary thyroid cancer through targeting BRAF	(67)	2D and PDX
Steroid receptor coactivator-1 interacts with NF-κB to increase VEGFC levels in human thyroid cancer	(68)	2D and PDX
MicroRNA-361-5p inhibits papillary thyroid carcinoma progression by targeting ROCK1	(69)	2D and PDX
INAVA promotes aggressiveness of papillary thyroid cancer by upregulating MMP9 expression	(70)	2D and PDX
LncRNA SNHG12 promotes the proliferation and metastasis of papillary thyroid carcinoma cells through regulating wnt/β-catenin signaling pathway	(71)	2D and PDX
LARP7 in papillary thyroid carcinoma induces NIS expression through suppression of the SHH signaling pathway	(72)	2D
miR-622 suppresses tumor formation by directly targeting VEGFA in papillary thyroid carcinoma	(73)	2D and PDX
Epigenetic Modifications in Thyroid Cancer Cells Restore NIS and Radio-Iodine Uptake and Promote Cell Death	(74)	2D
NEAT1_2 functions as a competing endogenous RNA to regulate ATAD2 expression by sponging microRNA-106b-5p in papillary thyroid cancer	(75)	2D
Histone methyltransferase KMT5A gene modulates oncogenesis and lipid metabolism of papillary thyroid cancer *in vitro*	(76)	2D
TBX3 promotes proliferation of papillary thyroid carcinoma cells through facilitating PRC2-mediated p57KIP2 repression	(77)	2D and PDX
Effects of miR-144 on the sensitivity of human anaplastic thyroid carcinoma cells to cisplatin by autophagy regulation	(78)	2D and PDX
Src-mediated regulation of the PI3K pathway in advanced papillary and anaplastic thyroid cancer	(79)	2D
Long non-coding RNA CCAL promotes papillary thyroid cancer progression by activation of NOTCH1 pathway	(80)	2D and PDX
Long glucocorticoid-induced leucine zipper regulates human thyroid cancer cell proliferation	(81)	2D and PDX
A dual mechanism of activation of the Sonic Hedgehog pathway in anaplastic thyroid cancer: crosstalk with RAS-BRAF-MEK pathway and ligand secretion by tumor stroma	(82)	2D and spheroids
Downregulation of CSN6 attenuates papillary thyroid carcinoma progression by reducing Wnt/β-catenin signaling and sensitizes cancer cells to FH535 therapy	(83)	2D and PDX
Long noncoding RNA NEAT1 regulate papillary thyroid cancer progression by modulating miR-129-5p/KLK7 expression	(84)	2D and PDX
VASN promotes YAP/TAZ and EMT pathway in thyroid carcinogenesis *in vitro*	(85)	2D
Knockdown of KDM1A suppresses tumor migration and invasion by epigenetically regulating the TIMP1/MMP9 pathway in papillary thyroid cancer	(86)	2D and PDX
Knockdown of long noncoding RNA SNHG7 inhibits the proliferation and promotes apoptosis of thyroid cancer cells by downregulating BDNF	(87)	2D
IGFBP7 inhibits cell proliferation by suppressing AKT activity and cell cycle progression in thyroid carcinoma	(88)	2D and PDX
AXL Is a Novel Predictive Factor and Therapeutic Target for Radioactive Iodine Refractory Thyroid Cancer	(89)	2D
Methylglyoxal Acts as a Tumor-Promoting Factor in Anaplastic Thyroid Cancer	(90)	2D
Functional analysis and clinical significance of the isocitrate dehydrogenase 2 gene in papillary thyroid carcinoma	(91)	2D
NECTIN4 promotes papillary thyroid cancer cell proliferation, migration, and invasion and triggers EMT by activating AKT	(92)	2D
TFAP2B overexpression contributes to tumor growth and progression of thyroid cancer through the COX-2 signaling pathway	(93)	2D and PDX
Down-regulated HSDL2 expression suppresses cell proliferation and promotes apoptosis in papillary thyroid carcinoma	(94)	2D and PDX
Mortalin (GRP75/HSPA9) Promotes Survival and Proliferation of Thyroid Carcinoma Cells	(95)	2D
LncRNA FOXD2-AS1 Functions as a Competing Endogenous RNA to Regulate TERT Expression by Sponging miR-7-5p in Thyroid Cancer	(96)	2D, spheroids and PDX
Interaction of BRAF-induced ETS factors with mutant TERT promoter in papillary thyroid cancer	(97)	2D and spheroids
Role of phospho–ezrin in differentiating thyroid carcinoma	(98)	2D
Low metallothionein 1M (MT1M) is associated with thyroid cancer cell lines progression	(99)	2D
MiR-758-3p regulates papillary thyroid cancer cell proliferation and migration by targeting TAB1	(100)	2D
RET Kinase-Regulated MicroRNA-153-3p Improves Therapeutic Efficacy in Medullary Thyroid Carcinoma	(101)	2D and PDX
Identification and characterization of two novel oncogenic mTOR mutations	(102)	2D and PDX
The Highly Expressed FAM83F Protein in Papillary Thyroid Cancer Exerts a Pro-Oncogenic Role in Thyroid Follicular Cells	(103)	2D and PDX
High expression of NUCB2 promotes papillary thyroid cancer cells proliferation and invasion	(104)	2D and PDX
miR-215 suppresses papillary thyroid cancer proliferation, migration, and invasion through the AKT/GSK-3β/Snail signaling by targeting ARFGEF1	(105)	2D and PDX
circRAPGEF5 Contributes to Papillary Thyroid Proliferation and Metastatis by Regulation miR-198/FGFR1	(106)	2D and PDX
Loss of MADD expression inhibits cellular growth and metastasis in anaplastic thyroid cancer	(107)	2D and PDX
Dual Oncogenic/Anti-Oncogenic Role of PATZ1 in FRTL5 Rat Thyroid Cells Transformed by the Ha-Ras^*V*12^ Oncogene	(108)	2D, spheroids and PDX
STAT3-induced upregulation of lncRNA ABHD11-AS1 promotes tumor progression in papillary thyroid carcinoma by regulating miR-1301-3p/STAT3 axis and PI3K/AKT signaling pathway	(109)	2D and PDX
SNHG15 functions as a tumor suppressor in thyroid cancer	(110)	2D
A Toxicogenomic Approach Reveals a Novel Gene Regulatory Network Active in *in vitro* and *in vivo* Models of Thyroid Carcinogenesis	(111)	2D and PDX
Downregulation of NEAT1 reverses the radioactive iodine resistance of papillary thyroid carcinoma cell via miR-101-3p/FN1/PI3K-AKT signaling pathway	(112)	2D and PDX
Silencing of lncRNA LINC00514 inhibits the malignant behaviors of papillary thyroid cancer through miR-204-3p/CDC23 axis	(113)	2D and PDX
MicroRNA-1270 modulates papillary thyroid cancer cell development by regulating SCAI	(114)	2D and PDX
TBX1 Functions as a Tumor Suppressor in Thyroid Cancer Through Inhibiting the Activities of the PI3K/AKT and MAPK/ERK Pathways	(115)	2D and PDX
MicroRNA-146b-5p as an oncomiR promotes papillary thyroid carcinoma development by targeting CCDC6	(116)	2D and PDX
KAT5 promotes invasion and metastasis through C-MYC stabilization in ATC	(117)	2D and PDX
MicroRNA-766 inhibits papillary thyroid cancer progression by directly targeting insulin receptor substrate 2 and regulating the PI3K/Akt pathway	(118)	2D and PDX
HOXD-AS1 is a predictor of clinical progression and functions as an oncogenic lncRNAs in papillary thyroid cancer	(119)	2D
MiR-141-3p Suppresses Tumor Growth and Metastasis in Papillary Thyroid Cancer via Targeting Yin Yang 1	(120)	2D and PDX
Long non-coding RNA LINC00152 promotes cell growth and invasion of papillary thyroid carcinoma by regulating the miR-497/BDNF axis	(121)	2D and PDX

* *PDX, patient derived xenografts, in vivo models implanting human cancer cells into immunodeficient mice*.

An important feature of these epigenetic studies is the ability to reverse the effects on gene expression and observe phenotypic characteristics of the cells. The general experimental methodology described in the studies begins with collection of human thyroid cancer specimens taken at the time of surgery and extraction of the messenger RNA. Real-time polymerase chain reaction (RT-PCR) is then performed on both the malignant and adjacent healthy thyroid tissue to compare the epigenetic profiles. Following this, established thyroid cancer cell lines are manipulated to replicate the particular expression profile identified in the human tissue samples. These cell lines are then cultured *in vitro* as monolayers and/or spheroids to examine phenotypic characteristics such as proliferation (e.g., Ki67 staining), cell viability and apoptosis (e.g., TUNEL assay), migration and invasion (e.g., wound-closure and Transwell chamber assays).

The study of cell migration in thyroid cancer research is essential as metastatic dissemination is a significant prognostic factor. For 2D monolayer culture systems, the wound-closure and the transwell migration/invasion assays are widely used. These assays can be applied to study the migratory ability of a whole cell mass but also an individual cell's morphology (122–125). Thyroid cancers that are known to have an aggressive phenotype can be studied for morphological features such asb invadopodia (126).

Alternatively, thyroid cancer cells can be completely immersed into a 3D matrix—either as a single cell suspension or more commonly as a spheroid (122, 125). This allows cells to migrate away from the tumor mass in any direction. The extent of migration/invasion is monitored at set intervals over the course of several days. This technique offers the benefit of performing the assay without having to re-plate the cells, as well as more closely simulating cell migration from a tumor mass *in vivo*. The oxygen and nutrient diffusion gradient present within a spheroid structure can promote migration and invasion through changes in gene expression—not present in 2D culture models. The effect of co-cultured cells such as macrophages on migration and invasion of thyroid cancer cells has also been explored as these immune cells have roles in epithelial-mesenchymal transition and subsequent tumor progression (14, 15). These assays are also applied in research studies testing the cytotoxic effects of chemotherapy agents.

Although no single genetic/epigenetic change has been reported, in all cases of thyroid cancer there are common signaling pathways which are affected. The epigenetic factors examined in the studies listed in Table 1 were shown to have either tumor suppressive or oncogenic effects via these pathways. Suppression of the PI3K/Akt pathway through silencing of TEKT4 (59), lncRNA XIST (55), miRNA-222 (64), LRP4 (63), and NECTIN 4 (92) led to reduced cellular proliferation and migration. Similarly, PI3K/Akt suppression and subsequent reduced tumorigenesis was achieved by up-regulating LncRNA-LINC003121 (40), miRNA-218 (49), miRNA-34a (55), and IGFBP7 (88). Up-regulation of miRNA-153-3p (101) and silencing of lncRNA-BANCR (62), TERT (97), and FAM83F (103) led to suppression of the MAPK/ERK pathway resulting in reduced cell proliferation and increased apoptosis. Silencing oncogenes SDC4 (51), lncRNA-UCA1 (46), lncRNA-SNHG12 (71), CSN6 (83) led to decreased proliferation and invasion, and increased apoptosis through inhibition of Wnt/b-catenin pathway, whereas up-regulating miRNA-329 (47) demonstrated the same effect.

The conclusion is that epigenetic molecules have the potential to be used as biomarkers as well as targets for drug therapy. The observation that thyroid cancer progression is associated with an accumulation of epigenetic changes has led to the development of drug treatments targeting these pathways such as multi-targeted tyrosine kinase inhibitors (TKIs), demethylating agents and histone deacetylase inhibitors (HDACi).

### Drug Testing

Drug attrition rates for cancer are much higher than in other therapeutic areas. Only 5% of agents that have anticancer activity in preclinical development demonstrate a sufficient efficacy in phase III testing (127). Although surgery and RAI therapy are primary therapeutic modalities for all subtypes of thyroid cancer, targeted drug therapies such as the tyrosine kinase inhibitors (TKIs) vemurafenib, sunitinib and lenvatinib are available for those patients with either rapidly progressing, recurrent or RAI-resistant thyroid cancers (128). Recent studies using *in vitro* models have focused on testing drug combinations to enhance tumor sensitivity to established chemotherapy agents, as well as testing novel anticancer agents and drug delivery systems (Table 2).

**Table 2:** Studies related to targeted drug testing in thyroid cancer research published from Jan 2018 to Jun 2019 (as per HDAS search on 19 July 2019).

**Publication title**	**References**	**Models used**
Precision Targeted Therapy with BLU-667 for RET -Driven Cancers	(129)	2D and PDX
The mTOR Kinase Inhibitor CZ415 Inhibits Human Papillary Thyroid Carcinoma Cell Growth	(130)	2D and PDX
Triple action Pt(iv) derivatives of cisplatin: a new class of potent anticancer agents that overcome resistance	(131)	2D, spheroids and PDX
Targeting of the Cholecystokinin-2 Receptor with the Minigastrin Analog 177Lu-DOTA-PP-F11N: Does the Use of Protease Inhibitors Further Improve *in vivo* Distribution?	(132)	2D
Recombinant oncolytic Newcastle disease virus displays antitumor activities in anaplastic thyroid cancer cells	(133)	2D, spheroid and PDX
The Synergistic Effects of Celecoxib and Sodium Valproate on Apoptosis and Invasiveness Behavior of Papillary Thyroid Cancer Cell Line *in-vitro*	(134)	2D
Metformin Targets Mitochondrial Glycerophosphate Dehydrogenase to Control Rate of Oxidative Phosphorylation and Growth of Thyroid Cancer *in vitro* and *in vivo*	(135)	2D and PDX
Selective RET kinase inhibition for patients with RET-altered cancers	(136)	2D and PDX
Potential of the dual mTOR kinase inhibitor AZD2014 to overcome paclitaxel resistance in anaplastic thyroid carcinoma	(137)	2D, spheroid and PDX
Heme Oxygenase-1 Inhibitors Induce Cell Cycle Arrest and Suppress Tumor Growth in Thyroid Cancer Cells	(138)	2D and PDX
Combined effects of octreotide and cisplatin on the proliferation of side population cells from anaplastic thyroid cancer cell lines	(139)	2D and PDX
The LAT1 inhibitor JPH203 reduces growth of thyroid carcinoma in a fully immunocompetent mouse model	(140)	2D and PDX
Synergistic effects of BET and MEK inhibitors promote regression of anaplastic thyroid tumors	(141)	2D and PDX
Apatinib-induced protective autophagy and apoptosis through the AKT–mTOR pathway in anaplastic thyroid cancer	(142)	2D and PDX
SoLAT (Sorafenib Lenvatinib alternating treatment): a new treatment protocol with alternating Sorafenib and Lenvatinib for refractory thyroid Cancer	(143)	2D and PDX
S100A4 Knockout Sensitizes Anaplastic Thyroid Carcinoma Cells Harboring BRAF V600E/Mt to Vemurafenib	(144)	2D and PDX
Lestaurtinib is a potent inhibitor of anaplastic thyroid cancer cell line models	(145)	2D and PDX
Computational modeling reveals MAP3K8 as mediator of resistance to vemurafenib in thyroid cancer stem cells	(146)	2D and spheroids
Emodin suppresses angiogenesis and metastasis in anaplastic thyroid cancer by affecting TRAF6-mediated pathways *in vivo* and *in vitro*	(147)	2D and PDX
PI3K blockage synergizes with PLK1 inhibition preventing endoreduplication and enhancing apoptosis in anaplastic thyroid cancer	(148)	2D and PDX
Transferrin receptor-targeted HMSN for sorafenib delivery in refractory differentiated thyroid cancer therapy	(149)	2D and PDX
Anti-hTERT siRNA-Loaded Nanoparticles Block the Growth of Anaplastic Thyroid Cancer Xenograft	(150)	2D and PDX
Melatonin suppresses thyroid cancer growth and overcomes radioresistance via inhibition of p65 phosphorylation and induction of ROS	(151)	2D and PDX
Vandetanib has antineoplastic activity in anaplastic thyroid cancer, *in vitro* and *in vivo*	(152)	2D and PDX
Lenvatinib exhibits antineoplastic activity in anaplastic thyroid cancer *in vitro* and *in vivo*	(153)	2D and PDX
Dual effects for lovastatin in anaplastic thyroid cancer: the pivotal effect of transketolase (TKT) on lovastatin and tumor proliferation	(154)	2D
*In vitro* Antitumor Activity of Aloperine on Human Thyroid Cancer Cells through Caspase-Dependent Apoptosis	(155)	2D
Epigenetic Modifications in Thyroid Cancer Cells Restore NIS and Radio-Iodine Uptake and Promote Cell Death	(74)	2D
Novel design of NIR-triggered plasmonic nanodots capped mesoporous silica nanoparticles loaded with natural capsaicin to inhibition of metastasis of human papillary thyroid carcinoma B-CPAP cells in thyroid cancer chemo-photothermal therapy	(156)	2D
Inhibition of mitochondrial respiration by tigecycline selectively targets thyroid carcinoma and increases chemosensitivity	(157)	2D and PDX
Targeting PLKs as a therapeutic approach to well-differentiated thyroid cancer	(158)	2D and PDX
Transcript-level regulation of MALAT1-mediated cell cycle and apoptosis genes using dual MEK/Aurora kinase inhibitor “BI-847325” on anaplastic thyroid carcinoma	(159)	2D and spheroid
Effect of Nifuroxazide on Proliferation, Migration, and Invasion of Thyroid Papillary Carcinoma Cells	(160)	2D
Antitumor Effect of ^131^I-Labeled Anti-VEGFR2 Targeted Mesoporous Silica Nanoparticles in Anaplastic Thyroid Cancer	(161)	2D and PDX
MAPK Inhibitors Enhance HDAC Inhibitor-Induced Redifferentiation in Papillary Thyroid Cancer Cells Harboring BRAF V600E: An *in vitro* Study	(162)	2D
Evaluation of preclinical efficacy of everolimus and pasireotide in thyroid cancer cell lines and xenograft models	(163)	2D and PDX
Propofol suppresses proliferation and migration of papillary thyroid cancer cells by down-regulation of lncRNA ANRIL	(164)	2D
Discovery of Potent, Selective, and Orally Bioavailable Estrogen-Related Receptor-γ Inverse Agonists To Restore the Sodium Iodide Symporter Function in Anaplastic Thyroid Cancer	(165)	2D
Antitumor effects of anlotinib in thyroid cancer	(166)	2D and PDX

Nanontechnology, and more specifically manufacture of nanoparticles for drug formulation and delivery has been a promising area of research. Drug products that contain proteins or nucleic acids are susceptible to pharmacokinetic degradation. Nanoparticles can be customized for targeted delivery of drugs to improve bioavailability and provide controlled release of medication (167). For thyroid cancers, nanoparticles have been used to deliver sorafenib (149), anti-hTERT siRNA (150), capsacin for use in photothermal therapy (156), and I^131^ labeled anti-VEGFR2 antibodies for targeted drug delivery and have shown promising results in both *in vitro* and *in vivo* cancer models.

As with the epigenetic studies, most drug studies utilize a combination of primary human thyroid cancer cells and established cell lines. Cells are cultured in monolayers and spheroids and exposed to incremental drug levels to observe cell survival and apoptosis. Thyroid cancer cells are also injected into animal models (patient derived xenografts—PDX) and treated with drugs to validate *in vitro* findings.

3D culture models are emerging as improved experimental models for preclinical target identification. Although spheroids more closely resemble the tumor *in vivo*, there is currently no commercially available standardized high-throughput assay for drug screening. Earlier studies by Li et al. (168), Guiffrida et al. (169), Hardin et al. (170) compared drug sensitivities on various types of thyroid cancer grown in 2D and 3D culture systems. They all observed that drug resistance was much higher in cells that formed spheroids than in monolayers. These findings have been attributed to the diffusion dynamics seen in spheroids as well as the discovery of side populations of cells within tumors that demonstrate stem cell-like properties.

### Cancer Stem Cells

Cancer stem cell (CSC) theory challenges the classical model of carcinogenesis (where any cell in an organ has the potential to transform through gene mutations) following the discovery of distinct populations of pluripotent tumor stem-like cells within solid tumors (171). In thyroid cancer, small populations of cells within a tumor have displayed distinct CD surface antigens and gene expression (e.g., Oct 3 and 4, Nanog) that are known to be associated with stem cells identified in other forms of cancer (172) (Table 3).

**Table 3:** Studies related to thyroid cancer stem cells published from 2008 to 2019 (as per HDAS search on 19 July 2019).

**Publication title**	**References**	**Models used**
*In vitro* identification and characterization of CD133(pos) cancer stem-like cells in anaplastic thyroid carcinoma cell lines	(173)	2D
Medullary Thyroid Carcinoma Cell Lines Contain a Self-Renewing CD133 Population that Is Dependent on Ret Proto-Oncogene Activity	(174)	2D and spheroids
Tumorigenic and Metastatic Activity of Human Thyroid Cancer Stem Cells	(175)	2D, spheroid and PDX
Doxorubicin fails to eradicate cancer stem cells derived from anaplastic thyroid carcinoma cells: characterization of resistant cells	(176)	2D and spheroids
Insulin receptor isoforms and insulin-like growth factor receptor in human follicular cell precursors from papillary thyroid cancer and normal thyroid	(177)	2D and spheroids
Phenotypic Characterization of Metastatic Anaplastic Thyroid Cancer Stem Cells	(168)	2D, spheroid and PDX
Detection of Thyroid Cancer Stem Cells in Papillary Thyroid Carcinoma	(53)	2D, spheroid and PDX
SNAIL induces epithelial-to-mesenchymal transition and cancer stem cell-like properties in aldehyde dehydroghenase-negative thyroid cancer cells	(178)	2D, spheroid and PDX
Analysis of multiple markers for cancer stem-like cells in human thyroid carcinoma cell lines	(179)	2D and spheroids
Stemness in Human Thyroid Cancers and Derived Cell Lines: The Role of Asymmetrically Dividing Cancer Stem Cells Resistant to Chemotherapy	(180)	2D and PDX
Thyrospheres From Normal or Malignant Thyroid Tissue Have Different Biological, Functional, and Genetic Features	(181)	Spheroids
Molecular profiles of cancer stem-like cell populations in aggressive thyroid cancers	(182)	2D and spheroids
Resistance of papillary thyroid cancer stem cells to chemotherapy	(169)	Spheroids
Generation of Novel Thyroid Cancer Stem-Like Cell Clones	(170)	2D, spheroid and PDX
Intracellular redox status controls spherogenicity, an *in vitro* cancer stem cell marker, in thyroid cancer cell lines	(183)	2D and spheroids
Thyroid Cancer Stem-Like Cell Exosomes: Regulation of EMT via Transfer of LncRNAs	(184)	2D and spheroids
β-catenin nuclear translocation represses thyroid cancer stem cells differentiating into cells with sodium-iodine symporter functional expression	(185)	2D and PDX
Effect of low-dose tungsten on human thyroid stem/precursor cells and their progeny	(186)	2D and spheroid

Generally, the studies have used *in vitro* sphere formation assays and PDX (using immunedeficient murine models) to confirm the existence of CSC. In 2007, Mitsutake et al., were the first group to identify and characterize a very small side population of putative thyroid cancer stem cells (CSC; 0.02–0.25% of total number of cells) from thyroid cancer cell lines (187). The highest percentage of CSC was seen in anaplastic thyroid cancer cell lines (ATC). The CSC in this study demonstrated stem-like properties of self-renewal and differentiation potential as well as altered gene expression profiles compared with non-CSC cells.

Since then more researchers have used *in vitro* models to identify specific tumor markers in thyroid CSC previously validated in other types of cancer. High levels of Oct-4, SOX-2, NANOG, and CD44 have been associated with thyroid CSC (53, 168, 170, 177). Conversely, the cells isolated in these studies expressed low or completely absent levels of thyroid-specific differentiation markers such as TTF1, PAX8, and TSH-R.

When proliferation, migration, and cell survival assays have been applied to CSC *in vitro* they have demonstrated increased metastatic potential and reduced apoptosis (170, 172, 182, 188). Additionally, they are largely quiescent which allows them to escape chemotherapy agents that normally target rapidly dividing cells (169).

## Concluding Statement and Future Perspective

In this review we have established that *in vitro* cell culture models have been the workhorse in thyroid cancer research for decades. There have been many advances in culture techniques- developing complex cellular architecture that more closely resemble tumors *in vivo*.

*In vitro* culture models have provided researchers with a reliable platform to study the molecular and cellular biology of thyroid cancer, as well as for testing drugs prior to human trials. In the future, the promising field of personalized cancer medicine will establish effective treatment strategies based on an individual tumor's genetic profile and predicted drug response through *in vitro* culture techniques.

## Author Contributions

DC was the primary author of this article. VG and JG also reviewed articles that were included in the literature review. AR, RE, VG, and JG contributed to proof reading and editing of the final version. All authors contributed to the article and approved the submitted version.

## Conflict of Interest

The authors declare that the research was conducted in the absence of any commercial or financial relationships that could be construed as a potential conflict of interest.
